# On the Bayesian generalized extreme value mixture autoregressive model with adjusted SNR in non-standard actuarial data

**DOI:** 10.1016/j.mex.2024.103095

**Published:** 2024-12-09

**Authors:** Chrisandi R. Lande, Nur Iriawan, Dedy Dwi Prastyo

**Affiliations:** a Department of Statistics, Institut Teknologi Sepuluh Nopember, Surabaya 60111 Indonesia; b Politeknik Ilmu Pelayaran Makassar, Makassar 90165, Indonesia

**Keywords:** GEVMAR, Bayesian method, Time series, Adjusted SNR, Claim reserves, Bayesian Generalized Extreme Value Mixture Autoregressive

## Abstract

This research introduces the Generalized Extreme Value Mixture Autoregressive (GEVMAR) model as an innovative approach for examining non-standard actuarial datasets within general insurance. Information concerning claim reserves often reveals notable volatility and multimodal distributions, attributes that standard models, including previous method such as the Gaussian Mixture Autoregressive (GMAR) model and other autoregressive methodologies, find problematic to manage effectively. The GEVMAR model integrates the Generalized Extreme Value (GEV) distribution alongside Bayesian estimation techniques, augmented by a modified Signal-to-Noise Ratio (SNR) metric to improve predictive accuracy. Compared to preceding studies that adopted Gaussian-based or more elementary autoregressive models, the GEVMAR model displays a significantly elevated capacity to interpret complex data dynamics. The effectiveness of this methodological advancement has been rigorously assessed through its implementation to claim reserves data from insurance companies in Indonesia covering the period from 2015 to 2023, demonstrating that the GEVMAR model (GEV type I) consistently attains an improved adjusted SNR metric (1.3894 × 10⁶) coupled with a reduced Mean Absolute Percentage Error (MAPE) (0.0189) when compared to the GMAR model (MAPE 7.5812). Furthermore, the Bayesian methodology employed within the GEVMAR framework affords substantial versatility in incorporating prior distributions, thereby conferring a pivotal advantage in analyzing heavy-tailed datasets characterized by extreme variability. This study emphasizes the limitations of existing models, such as their reduced accuracy in capturing multimodal patterns and inability to address extreme volatility effectively. Some highlights of the proposed method are:

• Development of a new model for the generalized extreme value mixture autoregressive.
• Adjustment of SNR type 2 for the generalized extreme value mixture autoregressive model.
• Application of the Bayesian GEVMAR (GEV type I) model to non-standard claim reserves data.

## Graphical abstract

**Figure:**
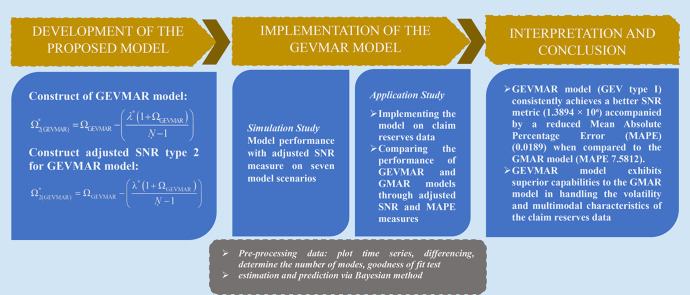

Specifications table

**Table:** 

Subject area:	Mathematics and Statistics
More specific subject area:	Statistics, time series model, Bayesian Analysis
Name of your method:	Bayesian Generalized Extreme Value Mixture Autoregressive
Name and reference of original method:	Original Method References: • P. de Jong and B. Zehnwirth, “Claim Reserving, State Space Models and the Kalman Filter,” J. Inst. Actuar., pp. 157–181, 1983. • Wong, C. S., & Li, W. K. (2000). On a mixture autoregressive model. Journal of the Royal Statistical Society: Series B (Statistical Methodology), 62(1), 95–115. • Ravagli, D., & Boshnakov, G. N. (2022). Bayesian analysis of mixture autoregressive models covering the complete parameter space. Computational Statistics, 37(3), 1399–1433
Resource availability:	The data used is general insurance claim reserves data in Indonesia from January 2015 to June 2023 sourced from Otoritas Jasa Keuangan (OJK), Republic of Indonesia.

## Background

Development of stochastic methods for estimating the value of claim reserves or Incurred But Not Reported (IBNR) is becoming widespread [1]. Additionally, these methods are divided into two namely frequency-static and Bayesian-dynamic. The frequency-static method uses the results of the Chain Ladder, Bournhutter-Ferguson, and Cape Cod. In 1985, Hertig [2] applied the Chain Ladder process with a lognormal age-to-age factor. Subsequently, Thomas Mack [3] used the Maximum Likelihood Estimation (MLE) with gamma distribution to estimate the IBNR of the Chain Ladder process.

From 1976 to 1981, Benktander-Hovinen Method [4,5] considered the forerunner of the Bayesian principle adopted prior estimates from the Chain Ladder and Bornhuetter-Ferguson processes, resulting in an exceptional performance [6]. Subsequently, Bayesian analysis was effectively conducted on insurance data by preliminary research [[7], [8], [9]]. Jackie Li [10] compared several stochastic models of incremental claim payment data, and reported that the Bayesian method formulated by Ntzoufras and Dellaportas [8] was more effective than the others.

Several stochastic methods for estimating claim reserves are not based on time series models, despite being in this form. The aggregate data, either from claim payments or incurred, could not always be formed into a run-off triangle, prompting de Jong and Zehnwirth [11] to lay the foundation for the formation of time series data. This idea led to the development of a non-run-off triangle data. Furthermore, time series models for non-run-off triangle data was generated by Cummins & Griepentrog, Kartikasari & Imani, and Kumar et al. [[12], [13], [14]] using the ARIMA to predict the size of general insurance claims or reserves.

General insurance data considered as long tail business including claim payments, number of claims incurred, and reserves, contain several distinct information that affect the value of claims [15,16]. This also has a significant impact on nonlinearity and multimodality, even though the acquired information was in the form of aggregate data [17]. Wong and Li [18] stated that specific nonlinear models are characterized by multimodal marginal distributions. Therefore, to address this issue, a Gaussian mixture autoregressive (GMAR) model was developed, with the expectation maximization algorithm used to estimate the parameters. Sampietro [19] used the Bayesian method to generate GMAR model, and the reversible jump MCMC (RJMCMC) to determine the autoregressive order in each component [20]. In addition, a complete Bayesian analysis of GMAR models from the selection to sampling process, comprised the shortcomings of the investigation conducted by Sampietro [21,22].

Taylor [23] focused on insurance data, using lognormal and gamma distribution to model both claim and delayed payments (open claims). Meanwhile, because insurance data is a long-tail business, it must consider the correct distribution assumptions that describes the number of claims to be paid immediately before an issue occurs leading to the financial instability of the company. According to Gomes and Pestana [24], some branches of the general insurance business, such as property, aviation, and shipping, tend to rule out outlier observation data patterns, characterized by extreme changes in claim values. Gomes and Pestana further suggested a generalized extreme value (GEV) distribution that can accommodate both heavy and light tail data patterns. The GEV distribution is suitable for general insurance claims data with large values, regarded as a relaxation of the normal, lognormal, and gamma distributions [24]. Bayesian methods used to evaluate mixture autoregressive models, multimodality features, and GEV distribution have been proven to present certain challenges when applied to general insurance claim reserves data in the form of time series. Therefore, these methods were adopted to construct the generalized extreme value mixture autoregressive (GEVMAR) model. Adjusted signal-to-noise ratio (SNR) was developed to assess the performance of the model using simulation. Moreover, this method can also be used to examine data regarding general insurance claim reserves.

## Method details

This study aims to develop a representative GEVMAR model to accurately predict the value of general insurance claim reserves in Indonesia. This section outlines the methodological framework used to construct the GEVMAR model, which employs the Bayesian generalized extreme value type I mixture autoregressive model or GEVMAR (GEV type I) model approach for estimation and prediction.

### Construction of generalized extreme value mixture autoregressive model

#### Generalized extreme value distribution

The GEV distribution is an assembly of continuous probability distributions resulting from the combination of extreme value theory with the Gumbel, Frèchet, and Weibull distributions. These three belonged to the types I, II, and III families of generalized extreme value distributions. The GEV distribution is defined by the cumulative distribution function (CDF), represented as

(1) $$H\left(y\right)=\left\{ \begin{array}{c} exp\left(-{\left(1+\xi \left(\frac{y-\mu }{\varsigma }\right)\right)}^{-\frac{1}{\xi }}\right),\xi \neq 0,\\ exp\left(-exp\left(-\left(\frac{y-\mu }{\varsigma }\right)\right)\right),\xi =0, \end{array}\right.$$

with μ,ς,ξ depicting the location, scale, and shape parameters. Furthermore, the probability density function (pdf) of the GEV distribution can be stated as follows:

(2) $$h\left(y|\mu ,\varsigma ,\xi \right)=\frac{1}{\varsigma }{\left(1+\xi \left(\frac{y-\mu }{\varsigma }\right)\right)}^{\left(-1/\xi \right)-1}exp\left(-{\left(1+\xi \left(\frac{y-\mu }{\varsigma }\right)\right)}^{\left(-1/\xi \right)}\right),$$

If ξ=0, The pdf is expressed in Eq. (3)

(3) $$h\left(y|\mu ,\varsigma ,0\right)=\frac{1}{\varsigma }exp\left(-\left(\frac{y-\mu }{\varsigma }\right)\right)exp\left(-exp\left(-\frac{y-\mu }{\varsigma }\right)\right).$$

#### Gaussian mixture autoregressive (GMAR) model parameter estimation

Reformulation of the GMAR model with diverse components contributed to the CDF and pdf of the GMAR model [18], expressed as follows:

(4) $$G\left(y_{t}|\Psi_{\left(t-1\right)}\right)=\sum_{r=1}^{R}\varpi_{r}\Lambda \left(\frac{y_{t}-\left({б}_{r.0}+\sum_{i=1}^{p_{r}}{б}_{r.i}y_{t-i}\right)}{\zeta_{r}}\right),$$

(5) $$\rho_{r.t}={б}_{r.0}+\sum_{i=1}^{p_{r}}{б}_{r.i}y_{t-i}$$

(6) $$g\left(y_{t}|\Psi_{\left(t-1\right)};{б}_{r.0},{б}_{r.p_{r}},\zeta_{r},\varpi_{r}\right)=\sum_{r=1}^{R}\frac{\varpi_{r}}{\zeta_{r}}\varphi \left(\frac{y_{t}-{б}_{r.0}-\sum_{i=1}^{p_{r}}{б}_{r.i}y_{t-i}}{\zeta_{r}}\right);t=1,2,..,T.$$

$\Psi_{\left(t-1\right)}$ denotes the set of information up to time t−1, Λ(·) depicts CDF of the normal distribution, and φ(·) denotes pdf of the normal distribution. $p_{r}$ is the autoregressive order of the r mixture components, $\zeta_{r}$ depicts the standard deviation of the r mixture components, with proportions $\sum_{r=1}^{R}\varpi_{r}=1,\varpi_{r}>0,$ and R denotes the number of mixture components. Meanwhile, the likelihood function of the GMAR$\left(R;p_{1},p_{2},\cdot \cdot \cdot ,p_{r}\right)$ model is stated as follows:

(7) $$L\left(\varphi ,\zeta_{r},\varpi_{r}|y,Z\right)=\prod_{t=p+1}^{n}\prod_{r=1}^{R}{\left(\frac{\varpi_{r}}{\zeta_{r}}\varphi \left(\frac{y_{t}-{б}_{r.0}-\sum_{i=1}^{p_{r}}{б}_{r.i}y_{t-i}}{\zeta_{r}}\right)\right)}^{Z_{t.r}}$$

Since $Z_{t.r}$ denotes a vector of unobservable variables, it is difficult to directly maximize the log-likelihood function. The problem was resolved by applying the expectation maximization (EM) algorithm [[18], [25]]. However, Ravagli and Boshnakov [22] used the Bayesian approach to estimate the GMAR model.

#### Generalized extreme value mixture autoregressive (GEVMAR) model

The GEVMAR model was developed from the GMAR aimed to accommodate the multimodal nature of GEV distributed time series data in non-standard actuary form.

Considering that the GMAR model and CDF of the GEV distribution were obtained using Eqs. (4) and (1), respectively. Therefore, both equations could be represented in this format

(8) $$G\left(y_{t}|\Psi_{\left(t-1\right)}\right)=\sum_{r=1}^{R}\varpi_{r},H_{\left(\xi_{r}\right)}\left(\frac{y_{t}-\mu_{r.t}}{\varsigma_{r}}\right),$$

or can be expressed

(9) $$y_{t}=\left\{ \begin{array}{c} \mu_{1.t}+\varsigma_{1}e_{1.t};\;\text{with}\;\text{probability}\;\varpi_{1};\\ \mu_{2.t}+\varsigma_{2}e_{2.t};\;\text{with}\;\text{probability}\;\;\varpi_{2};\\ \vdots \\ \mu_{R.t}+\varsigma_{R}e_{R.t};\;\text{with}\;\text{probability}\varpi_{R}, \end{array}\right.$$

with

(10) $$\mu_{r.t}={б}_{r.0}+\sum_{i=1}^{p_{r}}{б}_{r.i}y_{t-i}.$$

Supposedly alternative expression for the corrected mean was denoted by [21,22]

$$\begin{array}{ccc} \mu_{r.t} & = & \mu_{r}-\sum_{i=1}^{p_{r}}{б}_{r.i}\mu_{r}+\sum_{i=1}^{p_{r}}{б}_{r.i}y_{t-i}\\ & = & \mu_{r}\left(1-\sum_{i=1}^{p_{r}}{б}_{r.i}\right)+\sum_{i=1}^{p_{r}}{б}_{r.i}y_{t-i}, \end{array}$$

with ${б}_{r.0}=\mu_{r}\left(1-\sum_{i=1}^{p_{r}}{б}_{r.i}\right),$ and if $\sum_{i=1}^{p_{r}}{б}_{r.i}\neq 1$ then $\mu_{r}=\frac{{б}_{r.0}}{1-\sum_{i=1}^{p_{r}}{б}_{r.i}},$was obtained here r=1,2,···,R with R depicting the number of components, $\varpi_{R}$ denoting the weights or proportions with bounds $\varpi_{r}>0$, and $\sum_{r=1}^{R}\varpi_{r}=1$. ${}^{\prime }H_{\left(\xi_{r}\right)}\left(\cdot \right)$ represented CDF of the GEV distribution with conditional identities of the shape parameters $\xi_{r}\neq 0$, and $\xi_{r}=0$. While $\varsigma_{r}$ the scale parameter in each r mixture components, ${б}_{r.0}$ depicted the autoregressive (AR) constant of r mixture components, ${б}_{r.i}$ denoted the AR coefficient of r mixture components, $i=1,2,\cdot \cdot \cdot p_{r}$ where $p_{r}$ states the AR order of r mixture components. Therefore, the model can be represented as GEVMAR$\left(R;p_{1},p_{2},\cdot \cdot \cdot ,p_{r}\right)$ with parameters $\Theta ={\left(\theta ,\varpi \right)}^{\prime };$$\theta ={\left(\xi_{r},\varsigma_{r},\phi \right)}^{\prime };$$\varpi ={\left(\varpi_{1},\cdot \cdot \cdot ,\varpi_{R}\right)}^{\prime };$ where the AR coefficient $\phi ={\left({б}_{r.1},\cdot \cdot \cdot ,{б}_{r.p_{r}}\right)}^{\prime }$ was assumed to satisfy $1-\sum_{i=1}^{p_{r}}{б}_{r.i}{Đ}^{i}\neq 0$ for $|\sum_{i=1}^{p_{r}}{б}_{r.i}{Đ}^{i}|<1$, ${Đ}^{i}$ representing the backshift operator of ${Đ}^{i}y_{t}=y_{t-i}$. Therefore, Eqs. (8) and (9) were in stationary conditions for each AR model of r mixture components [[26], [27]].

**Proof:**

Based on the GMAR model in Eq. (4), the conditional pdf was expressed in Eq. (6) and $\rho_{r.t}$ in Eq. (5). Assuming the normal distributed conditional pdf in the GMAR model was substituted with the GEV distribution as in Eqs. (2) and (3), the GEVMAR conditional pdf is obtained as follows

(11) $$g\left(y_{t}|\Psi_{\left(t-1\right)}\right)=\sum_{r=1}^{R}\frac{\varpi_{r}}{\varsigma_{r}}\hbar {\left(y_{t}\right)}^{\xi_{r}+1}\exp\left(-\hbar \left(y_{t}\right)\right),$$

with

$$\hbar \left(y_{t}\right)=\left\{ \begin{array}{cc} 1+\xi_{r}{\left(\frac{y_{t}-\mu_{r.t}}{\varsigma_{r}}\right)}^{-1\;/\;\xi_{r}}, & \xi_{r}\neq 0,\\ \exp\left(-\left(\frac{y_{t}-\mu_{r.t}}{\varsigma_{r}}\right)\right), & \xi_{r}=0. \end{array}\right.$$

Assuming $h_{\left(\xi_{r}\right)}\left(\cdot \right)$ denotes the pdf of the GEV distribution, the pdf of the GEVMAR model can be expressed as follows

$$g\left(y_{t}|\Psi_{\left(t-1\right)};\Theta \right)=\sum_{r=1}^{R}\frac{\varpi_{r}}{\varsigma_{r}}h_{\left(\xi_{r}\right)}\left(\frac{y_{t}-\mu_{r.t}}{\varsigma_{r}}\right).$$

Assuming $H_{\left(\xi_{r}\right)}\left(\cdot \right)$ denotes the CDF symbol of the GEV distribution, then the CDF of the GEVMAR model can be written as follows

$$G\left(y_{t}|\Psi_{\left(t-1\right)};\Theta \right)=\sum_{r=1}^{R}\varpi_{r}H_{\left(\xi_{r}\right)}\left(\frac{y_{t}-\mu_{r.t}}{\varsigma_{r}}\right).$$

#### Estimation and prediction of GEVMAR (GEV type I) model using the Bayesian approach

In order to estimate the parameters in the GEVMAR model, we need to construct the likelihood function using equation (11) to proceed. Let $Y_{1},Y_{2},\ldots ,Y_{T}$ be a random variable with the conditional probability density function denoted as $g(y_{t}|\Psi_{\left(t-1\right)}).$ The probability function of the $\text{GEVMAR}(R;p_{1},p_{2},\cdot \cdot \cdot ,p_{r})$ model was expressed as the likelihood function.

$$\begin{array}{ccc} L\left(Y_{t},\ldots ,Y_{T}\right) & = & \prod_{t=p+1}^{n}g\left(y_{t}|\Psi_{\left(t-1\right)}\right).\\ & = & \prod_{t=p+1}^{n}\left(\sum_{r=1}^{R}\frac{\varpi_{r}}{\varsigma_{r}}\hbar {\left(y_{t}\right)}^{\xi_{r}+1}\exp\left(-\hbar \left(y_{t}\right)\right)\right). \end{array}$$

Let $Z_{t.r}$ be an unobservable random variable, taking the value of 1 assuming $y_{t}$ originated from the r mixture components and 0 otherwise. In order to restate the likelihood function of the GEVMAR model in improved conduct [[18], [25]], it stated as follows

(12) $$L\left(Y_{t},\ldots ,Y_{T}\right)=\prod_{t=p+1}^{n}\prod_{r=1}^{R}{\left(\frac{\varpi_{r}}{\varsigma_{r}}\hbar {\left(y_{t}\right)}^{\xi_{r}+1}\exp\left(-\hbar \left(y_{t}\right)\right)\right)}^{Z_{t.r}},$$

Therefore, the log-likelihood function is

$$\begin{array}{ccc} \text{log}L\left(y_{p+1},\ldots ,y_{n}\right) & = & \text{log}(\sum_{t=p+1}^{n}\sum_{r=1}^{R}\left(Z_{t.r}\frac{\varpi_{r}}{\varsigma_{r}}\hbar {\left(y_{t}\right)}^{\xi_{r}+1}\exp\left(-\hbar \left(y_{t}\right)\right)\right))\\ & = & \text{log}(\sum_{t=p+1}^{n}\sum_{r=1}^{R}Z_{t.r}\left(\varpi_{r}\left(\frac{1}{\varsigma_{r}}\hbar {\left(y_{t}\right)}^{\xi_{r}+1}\right)\exp\left(-\hbar \left(y_{t}\right)\right)\right)). \end{array}$$

Supposing $\text{log}L(y_{p+1},\ldots ,y_{n})=l,$ then

(13) $$l=\sum_{t=p+1}^{n}(\sum_{r=1}^{R}Z_{t.r}\text{log}\left(\varpi_{r}\right)-\sum_{r=1}^{R}Z_{t.r}\text{log}\left(\varsigma_{r}\right)+\sum_{r=1}^{R}Z_{t.r}\left(\xi_{r}+1\right)\text{log}\left(\hbar \left(y_{t}\right)\right)-\sum_{r=1}^{R}Z_{t.r}\left(\hbar \left(y_{t}\right)\right)).$$

Considering that $y_{t}$ was assumed to follow a GEV type I $\left(\text{GE}V_{I}\right)$ distribution, specifically the Gumbel distribution in the GEVMAR model, the following was derived:

(14) $$l=\sum_{t=p+1}^{n}(\sum_{r=1}^{R}Z_{t.r}\text{log}\left(\varpi_{r}\right)-\sum_{r=1}^{R}Z_{t.r}\text{log}\left(\varsigma_{r}\right)-\sum_{r=1}^{R}Z_{t.r}\left(\frac{y_{t}-\mu_{r.t}}{\varsigma_{r}}\right)+\sum_{r=1}^{R}Z_{t.r}\left(\exp\left(\frac{y_{t}-\mu_{r.t}}{\varsigma_{r}}\right)\right)),$$

with $\mu_{r.t}={б}_{r.0}+\sum_{i=1}^{p_{r}}{б}_{r.i}y_{t-i},$

Then supposing that

$$\begin{array}{ccc} e_{r.t} & = & y_{t}-\mu_{r.t}\\ & = & y_{t}-{б}_{r.0}-\sum_{i=1}^{p_{r}}{б}_{r.i}y_{t-i}, \end{array}$$

Eq. (14) develops into

(15) $$l=\sum_{t=p+1}^{n}(\sum_{r=1}^{R}Z_{t.r}\text{log}\left(\varpi_{r}\right)-\sum_{r=1}^{R}Z_{t.r}\text{log}\left(\varsigma_{r}\right)-\sum_{r=1}^{R}Z_{t.r}\left(\frac{e_{r.t}}{\varsigma_{r}}\right)+\sum_{r=1}^{R}Z_{t.r}\left(\exp\left(\frac{e_{r.t}}{\varsigma_{r}}\right)\right)).$$

Considering that $Z_{t.r}$ is an unseen latent variable vector, it becomes challenging to directly maximize the log-likelihood function, and this problem was addressed using the Bayesian method. According to Gamerman [28], the Bayesian method relies on the posterior distribution, obtained by multiplying the likelihood function with prior distribution. Appropriate prior distributions were adopted for the latent variables and model parameters in order to obtain the posterior distribution, as stated in previous research [19,21,22].

The prior distribution for each parameter in Eq. (12) was determined using a hierarchical structural method [[22], [29]]. Meanwhile, the prior distribution used in the GEVMAR ($\text{GE}V_{I}$) model was stated as follows

(16) $$\varpi ∼\text{Dirichlet}\left(d_{1},...,d_{R}\right),d_{1}=\cdot \cdot \cdot =d_{R}=1$$

(17) $$\mu_{r}∼\text{GE}V_{I}\left(\beta ,1/\kappa \right),r=1,2,\cdot \cdot \cdot ,R$$

(18) δ∼Gamma(a,b)

(19) $$\eta_{r}|\delta ∼\text{Gamma}\left(c,\delta \right),r=1,2,...,R$$

In this research, the Random Walk Metropolis algorithm to sample autoregressive parameters from the posterior distribution was adopted [22]. The hyperparameters for the Dirichlet prior distribution on the mixture weights (ϖ) was set to 1. Furthermore, $\mu_{r}$ was assigned a prior distribution, following the generalized extreme value type I $\left(\text{GE}V_{I}\right)$. This distribution was characterized by specific hyperparameters, namely β for the mean and κ for the precision. While δ is a prior following the gamma distribution with predefined hyperparameters a and b. Prior $\eta_{r}$ automatically follows the gamma distribution with hyperparameters c and δ. The value of $K_{y}=\text{max}\left(y\right)-\text{min}\left(y\right)$ was provided, representing a range of data set. Additionally, the following hyperparameters were established $\beta =\text{min}\left(y\right)+K_{y}/2,$$\kappa^{-1}=K_{y},$ $b=\frac{100a}{cK_{y}^{2}}=\frac{10}{K_{y}^{2}},$ with a=0.2 and c=2.

According to Ravagli and Boshnakov [22], the posterior distribution can be obtained using the following equation

(20) $$P\left(Z_{t.r}|y_{t},\Theta \right)=\frac{\frac{\varpi_{r}}{\varsigma_{r}}exp\left(-\left(\frac{e_{r.t}}{\varsigma_{r}}\right)\right)exp\left(exp\left(\frac{e_{r.t}}{\varsigma_{r}}\right)\right)}{\sum_{r=1}^{R}\left(\frac{\varpi_{r}}{\varsigma_{r}}exp\left(-\left(\frac{e_{r.t}}{\varsigma_{r}}\right)\right)exp\left(exp\left(\frac{e_{r.t}}{\varsigma_{r}}\right)\right)\right)},$$

(21) $$\varpi |\Theta_{-\varpi },y,Z∼\text{Dirichlet}\left(1+m_{1},...,1+m_{R}\right),$$

(22) $$\mu_{r}|\Theta_{-\mu_{r}},y,Z∼\text{GE}V_{I}\left(\frac{\eta_{r}m_{r}{\bar{e}}_{r}{Ь}_{r}+\beta \kappa }{\eta_{r}m_{r}{Ь}_{r}^{2}+\kappa },\frac{1}{\eta_{r}m_{r}{Ь}_{r}^{2}+\kappa }\right),$$

(23) $$\delta |\Theta_{-\delta },y,Z∼\text{Gamma}\left(a+Rc,b+\sum_{r=1}^{R}\eta_{r}\right),$$

(24) $$\eta_{r}|\Theta_{-\eta_{r}},y,Z∼\text{Gamma}\left(c+\frac{m_{r}}{2},\delta +\frac{1}{2}\sum_{t=p+1}^{n}e_{r.t}^{2}Z_{t.r}\right),$$

where r=1,···,R, $e_{r.t}=y_{t}-\mu_{r.t},$ $m_{r}=\sum_{t=p+1}^{n}Z_{t.r},$ ${Ь}_{r}=1-\sum_{i=1}^{p_{r}}{б}_{r.i},$ ${\bar{e}}_{r}=\frac{1}{m_{r}}\sum_{t=p+1}^{n}e_{r.t}Z_{t.r}.$

### Construction of adjusted signal-to-noise ratio (SNR) for GEVMAR model

#### Signal-to-noise ratio (SNR) and adjusted SNR

The SNR metric was used to assess the relative strength of a signal compared to the accompanying noise. This metric (Ω) was obtained using the following equation [[30], [31]]

(25) $$\begin{array}{c} \Omega =\frac{Var\left({\hat{y}}_{t}\right)}{\sigma_{e}^{2}}=\frac{V\left({\hat{y}}_{t}\right)}{З/И}=\frac{V\left({\hat{y}}_{t}\right)}{V\left(e_{t}\right)}, \end{array}$$

when $\sigma_{e}^{2}$ is the noise level, $V({\hat{y}}_{t})$ is the variance of ${\hat{y}}_{t}$ and denotes the signal strength, and $e_{t}=y_{t}-{\hat{y}}_{t}$ is the residual or noise.

SNR is defined as the ratio of residual variance to expected value variance. Box and Reid et al. [[32], [33]] had formulated the SNR differently than this research. Therefore, the following SNR (Ω) formulation was proposed [[31], [34]]

(26) $$\Omega =\frac{{Ŗ}^{2}}{З/И},$$

where, ${Ŗ}^{2}$ depicts the coefficient of determination, a measure of deviation or variance created in a model, И is the total sum of square, while З is the residual sum of square. Based on Eq. (26), the following was obtained

(27) $$\Omega =\frac{{Ŗ}^{2}}{1-{Ŗ}^{2}}.$$

Eq. (27) states the relationship between SNR and ${Ŗ}^{2}$. It can be an adjusted SNR that absorbs the advantage of${Ŗ}_{\left(adj\right)}^{2}$. Furthermore, the adjusted SNR types 1 and 2 were stated in the following equations [34]:

(28) $${\Omega^{*}}_{1}=\frac{{Ŗ}_{\left(adj\right)}^{2}}{1-{Ŗ}_{\left(adj\right)}^{2}}.$$

Alternatively, it can be written as:

(29) $${\Omega^{*}}_{1}=\Omega -\left(\frac{\lambda \left(1+\Omega \right)}{\underset{,}{N}-1}\right).$$

In Eq. (29), λ denotes the number of predictor variables, $\underset{,}{N}$ denotes sample size, and $\lambda^{*}$ represents the number of parameters in the model, thereby resulting in the following

(30) $${\Omega^{*}}_{2}=\Omega -\left(\frac{\lambda^{*}\left(1+\Omega \right)}{\underset{,}{N}-1}\right).$$

#### Adjusted SNR for GEVMAR model

The expression for ${\hat{y}}_{t}$ was derived from Eq. (9), specifically

(31) $$\begin{array}{ccc} {\hat{y}}_{t} & = & E\left[y_{t}|\Psi_{\left(t-1\right)}\right]\\ & = & \sum_{r=1}^{R}\varpi_{r}\left({б}_{r.0}+{б}_{r.i}y_{t-i}+\cdot \cdot \cdot +{б}_{r.p_{r}}y_{t-p_{r}}\right), \end{array}$$

hence,

(32) $$\begin{array}{ccc} E\left[{\hat{y}}_{t}\right] & = & \sum_{r=1}^{R}\varpi_{r}\left({б}_{r.0}+{б}_{r.1}E\left[y_{t-1}\right]+\cdot \cdot \cdot +{б}_{r.p_{r}}E\left[y_{t-p_{r}}\right]\right)\\ & = & \sum_{r=1}^{R}\varpi_{r}\left({б}_{r.0}+\tau \left({б}_{r.1}+\cdot \cdot \cdot +{б}_{r.p_{r}}\right)\right) \end{array}$$

where $\tau =E\left[y_{t-1}\right]=E\left[y_{t-p_{r}}\right]=\left\{ \begin{array}{c} \mu_{r.t}+\varsigma_{r}\left(\ell_{1}-1\right)/\xi_{r},\\ \mu_{r.t}+\varsigma_{r}ƛ,\\ \infty , \end{array}\begin{array}{c} \xi_{r}\neq 0,\xi_{r}<1\\ \xi_{r}=0\\ \xi_{r}\geq 1 \end{array}\right.$with $\ell_{1}=\Gamma \left(1-\xi_{r}\right)$, ƛ=0.5772... (Euler's constant).

Furthermore, the conditional variance of $y_{t}$ was stated as follows [18]:

(33) $$\begin{array}{cc} \text{Var}\left(y_{t}|\Psi_{\left(t-1\right)}\right) & =E\left[y_{t}^{2}|\Psi_{\left(t-1\right)}\right]-{\left(E\left[y_{t}|\Psi_{\left(t-1\right)}\right]\right)}^{2}\\ & =\sum_{r=1}^{R}\varpi_{r}\varsigma_{r}^{2}+\sum_{r=1}^{R}\varpi_{r}\mu_{r.t}^{2}-{\left(E\left[y_{t}|\Psi_{\left(t-1\right)}\right]\right)}^{2}\\ & =\sum_{r=1}^{R}\varpi_{r}\varsigma_{r}^{2}+\sum_{r=1}^{R}\varpi_{r}{\left({б}_{r.0}+{б}_{r.1}y_{t-1}+\cdot \cdot \cdot +{б}_{r.p_{r}}y_{t-p_{r}}\right)}^{2}\\ & \;-{\left(\sum_{r=1}^{R}\varpi_{r}\left({б}_{r.0}+{б}_{r.1}y_{t-1}+\cdot \cdot \cdot +{б}_{r.p_{r}}y_{t-p_{r}}\right)\right)}^{2}. \end{array}$$

Based on Eqs. (31), (32), and (33), the variance of ${\hat{y}}_{t}$ was stated as follows [35]:

(34) $$\begin{array}{cc} \text{Var}\left({\hat{y}}_{t}\right) & =E\left[{\hat{y}}_{t}^{2}\right]-{\left(E\left[{\hat{y}}_{t}\right]\right)}^{2}\\ & =E\left[{\left(E\left[y_{t}|\Psi_{\left(t-1\right)}\right]\right)}^{2}\right]-{\left(E\left[E\left[y_{t}|\Psi_{\left(t-1\right)}\right]\right]\right)}^{2}\\ & =E\left[{\left(\sum_{r=1}^{R}\varpi_{r}\left({б}_{r.0}+{б}_{r.1}y_{t-1}+\cdot \cdot \cdot +{б}_{r.p_{r}}y_{t-p_{r}}\right)\right)}^{2}\right]-{\left(E\left[\sum_{r=1}^{R}\varpi_{r}\left({б}_{r.0}+{б}_{r.1}y_{t-1}+\cdot \cdot \cdot +{б}_{r.p_{r}}y_{t-p_{r}}\right)\right]\right)}^{2}, \end{array}$$

supposing the conditional variance of the residuals was stated as follows,

(35) $$\text{Var}\left(e_{t}|\Psi_{\left(t-1\right)}\right)=E\left[{\left(y_{t}-{\hat{y}}_{t}\right)}^{2}|\Psi_{\left(t-1\right)}\right].$$

Therefore, the variance of the residuals can be obtained using Eq. (35)

(36) $$\text{Var}\left(e_{t}\right)=E\left[\text{Var}\left(e_{t}\right)|\Psi_{\left(t-1\right)}\right].$$

Adjusted SNR type 2 for GEVMAR was determined using Eq. (9) as stated in (30)

(37) $$\Omega_{2\left(\text{GEVMAR}\right)}^{*}=\Omega_{\text{GEVMAR}}-\left(\frac{\lambda^{*}\left(1+\Omega_{\text{GEVMAR}}\right)}{\underset{,}{N}-1}\right),$$

with a correction factor, $cf=\frac{\lambda^{*}\left(1+\Omega_{\text{GEVMAR}}\right)}{\underset{,}{N}-1}.$where, Eqs. (25), (34), and (36), can be used to express SNR for GEVMAR

(38) $$\Omega_{\text{GEVMAR}}=\frac{E\left[{\left(E\left[y_{t}|\Psi_{\left(t-1\right)}\right]\right)}^{2}\right]-{\left(E\left[E\left[y_{t}|\Psi_{\left(t-1\right)}\right]\right]\right)}^{2}}{E\left[\text{Var}\left(e_{t}\right)|\Psi_{\left(t-1\right)}\right]},$$

and $\lambda^{*}$ denotes the total GEVMAR parameter stated as follows,

(39) $$\lambda^{*}=\sum_{r=1}^{R}s\left(\theta_{R}\right)+\left(R-1\right),$$

where, s(·) represents the operator used to obtain the total parameters in $\theta_{R}=\left\{\varpi_{r},\varsigma_{r},{б}_{r.0},{б}_{r.1},\cdot \cdot \cdot ,{б}_{r.p_{r}}\right\},$ and R the number of mixture components.

## Measures of model performance

Essentially the most crucial function, closely associated with predictive modeling or any kind of time series forecasting, is the analysis of model performance. This may offer an approximate assessment of how successfully the model has encapsulated the underlying data trends. The study examined the Signal-to-Noise Ratio (SNR) alongside the Adjusted Signal-to-Noise Ratio (Adjusted SNR) metrics for GEVMAR model, while concurrently assessing the Mean Absolute Percentage Error (MAPE) and Root Mean Squared Error (RMSE), which are generally recognized as essential for evaluating model efficacy.

MAPE (Mean Absolute Percentage Error) denotes the average percentage deviation between predicted values and their corresponding actual counterparts. It yields a clear percentage that articulates, on average, the degree to which the predictions diverge from the actual values.

(40) $$\text{MAPE}=\frac{1}{T}\sum_{t=1}^{T}\left|\frac{y_{t}-{\hat{y}}_{t}}{y_{t}}\right|\times 100.$$

Root Mean Squared Error (RMSE) computes the square root of the mean of the squared differences between predicted values and actual observations. Given that the square root is employed, the resultant error retains the same unit as the original data. RMSE assigns greater significance to larger errors; thus, it may be particularly useful for illuminating substantial deviations in predictive accuracy.

(41) $$\text{RMSE}=\sqrt{\frac{1}{T}\sum_{t=1}^{T}{\left(y_{t}-{\hat{y}}_{t}\right)}^{2}}$$

## Method validation

### Simulation study

#### Simulation design

Simulations were conducted for the GEVMAR ($\text{GE}V_{I}$) model formed using Eq. (16). Additionally, the following stages are carried out during the process

1. The model used for simulation was GEVMAR(R;1,1)where the maximum R=2. Therefore, the GEVMAR model can be stated as follows:
  (42) $$\begin{array}{cc} g\left(y_{t}|\Psi_{\left(t-1\right)}\right) & =\sum_{r=1}^{R}\varpi_{r}g_{r}\left(y_{t}|\Psi_{\left(t-1\right)}\right)\\ & =\sum_{r=1}^{R}\varpi_{r}\left({б}_{r.0}+{б}_{r.1}y_{t-1}+\varepsilon_{r.t}\right), \end{array}$$
  with $\varpi_{1}=0.5,\varpi_{2}=0.5,$ ${б}_{1.1}=0.5,{б}_{2.1}=0.3,$$\varepsilon_{r.t}∼\text{GE}V_{I}\left(\mu_{r},\varsigma_{r},\xi_{r}\right);\xi_{r}=0;r=1,2.$
2. Generate $\varepsilon_{1.t},\varepsilon_{2.t}$ with the value ${б}_{r.0}=0,\mu_{r}=0,\varsigma_{1}=1.2,\varsigma_{2}=0.5,r=1,2$.
3. Calculate $g_{r}\left(y_{t}|\Psi_{\left(t-1\right)}\right)=\left({б}_{r.0}+{б}_{r.1}y_{t-1}+\varepsilon_{r.t}\right);r=1,2.$
4. Generate $\pi_{r.t}$ as $\pi_{r.t}∼\text{Mul}t_{R}\left(1,\varpi \right),$ using ϖ=(0.5,0.5); where $\pi_{r.t}$ represents a multinomially distributed with r=1,2 [36].
5. Calculate $y_{t}$ with the equation $y_{t}=\pi_{1.t}g_{1}\left(y_{t}|\Psi_{\left(t-1\right)}\right)+\pi_{2.t}g_{2}\left(y_{t}|\Psi_{\left(t-1\right)}\right).$
6. The next step was to generate 30, 50, 100, and 500 on steps 1-5, then estimate the parameters of the GEVMAR model using the Bayesian approach with seven GEVMAR ($\text{GE}V_{I}$) model scenarios designed differently for each scenario based on the order of the autoregressive model, the following scenario model table is presented:

**Table 1**

GEVMAR model simulation scenarios.

**Table:** 

Model scenario	Order	
	1st component	2nd component
1	1	1
2	2	2
3	3	3
4	4	4
5	1	2
6	1	3
7	1	4

7. Calculate SNR and Adjusted SNR type 2 from step (6), then determine the maximum value of Adjusted SNR from each scenario.
8. Repeat step (7) in 1000 replications.
9. Calculate average of SNR, average of adjusted SNR type 2, standard deviation, and probability results from step (8).

#### Simulation results

The following shows the results of the simulated seven GEVMAR model scenarios that had been determined. Based on the simulation findings, we provide the average SNR and the adjusted SNR in a tabular format, together with their respective standard deviations. Additionally, a probability table is provided to accurately delineate the attributes of the GEVMAR model in comparison to the empirical model.

The results of the simulation performed for the previously mentioned scenarios are detailed in Table 2, illustrating the average of SNR and adjusted SNR, along with the associated standard deviation. The information obtained is in line with the findings reported by Fajar et al. [34], that as the candidate model approaches the true model specification, the values of SNR and adjusted SNR type 2 become higher than the other candidate models for each sample size ($\underset{,}{N}$). This was also applicable to specific distributed time series models, particularly the GEVMAR ($\text{GE}V_{I}$) model. ${\bar{\Omega }}_{\text{GEVMAR}}$ and ${\bar{\Omega }}_{2(\text{GEVMAR})}^{*}$ values of GEVMAR(2;1,1) were more significant than those of the candidate models GEVMAR(2;2,2), GEVMAR(2;3,3), GEVMAR(2;4,4), GEVMAR(2;1,2), GEVMAR(2;1,3), and GEVMAR(2;1,4). This was because the GEVMAR(2;1,1) happens to be the true model with similar specifications. However, ${\bar{\Omega }}_{2(\text{GEVMAR})}^{*}$ value was consistently less than ${\bar{\Omega }}_{\text{GEVMAR}}$ in each of the candidate models. This was due to the dependence on the correction factor (cf), affected by the number of parameters and sample size. The standard deviation derived from SNR and adjusted SNR showed that the value of adjusted SNR was consistently less than that of SNR in all potential models. This depicted that ${\bar{\Omega }}_{2(\text{GEVMAR})}^{*}$ values have a narrower range of variation compared to ${\bar{\Omega }}_{\text{GEVMAR}}$. Furthermore, the result showed a significant correlation between SNR and adjusted SNR type 2, as ${\bar{\Omega }}_{\text{GEVMAR}}$ and ${\bar{\Omega }}_{2(\text{GEVMAR})}^{*}$ values generated increases, it tends to accurately represent the true model. Moreover, based on the simulation results, the probability of accurately identifying the characteristics of the GEVMAR model is comparable to that of the true model.

**Table 2:** Simulation results of SNR and adjusted SNR on GEVMAR ($\text{GE}V_{I}$) model.

Model	Sample size ($\underset{,}{N}$)							
	30		50		100		500	
	${\bar{\Omega }}_{\text{GEVMAR}}$	${\bar{\Omega }}_{2(\text{GEVMAR})}^{*}$	${\bar{\Omega }}_{\text{GEVMAR}}$	${\bar{\Omega }}_{2(\text{GEVMAR})}^{*}$	${\bar{\Omega }}_{\text{GEVMAR}}$	${\bar{\Omega }}_{2(\text{GEVMAR})}^{*}$	${\bar{\Omega }}_{\text{GEVMAR}}$	${\bar{\Omega }}_{2(\text{GEVMAR})}^{*}$
GEVMAR(2;1,1)	**6.81 × 10^8^** [1.58 × 10^10^]	**5.63 × 10^8^** [1.30 × 10^10^]	**7.21 × 10^9^** [2.09 × 10^11^]	**6.48 × 10^9^** [1.87 × 10^11^]	**3.59 × 10^11^** [9.97 × 10^12^]	**3.41 × 10^11^** [9.47 × 10^12^]	**5.62 × 10^8^** [9.80 × 10^9^]	**5.57 × 10^8^** [9.71 × 10^9^]
GEVMAR(2;2,2)	880.66 [4889.57]	667.8 [3709.3]	733.73 [4265.45]	628.8 [3656.1]	350.69 [3929.58]	325.8 [3651.7]	4.17 [5.88]	4.1 [5.8]
GEVMAR(2;3,3)	52.40 [172.59]	35.83 [119]	40.37 [191.30]	32.77 [156.2]	14.31 [80.29]	12.92 [73]	2.71 [1.63]	2.64 [1.6]
GEVMAR(2;4,4)	12.32 [27.66]	7.27 [17.17]	12.90 [49.01]	9.78 [38.01]	5.83 [14.06]	5.07 [12.50]	2.11 [1.37]	2.04 [1.34]
GEVMAR (2;1,2)	3616.2 [23025]	2867.8 [18260.9]	6317.9 [59523]	5544.2 [52234.7]	5486.5 [51380]	5153.9 [48265.9]	12.8 [38]	12.7 [37.5]
GEVMAR (2;1,3)	4198.67 [78589.7]	3184.96 [59619.8]	742.81 [5769.6]	636.55 [4945.3]	241.52 [1622.6]	224.37 [1507.9]	9.25 [14.5]	9.11 [14.2]
GEVMAR (2;1,4)	1963.61 [23708.5]	1421.65 [17168.26]	251.22 [1497.7]	210.04 [1253.17]	57.66 [143]	52.92 [131.48]	7.37 [8.4]	7.23 [8.26]

[.] states standard deviation of SNR and adjusted SNR, ${\bar{\Omega }}_{\text{GEVMAR}}$ denotes average of SNR, ${\bar{\Omega }}_{2(\text{GEVMAR})}^{*}$ denotes average of adjusted SNR type 2.

Table 3 illustrates a probability of the frequency of the best SNR and adjusted SNR values of all candidate models in each replication (1000 replications). Table 3 shows that candidate model 1 (GEVMAR(2;1,1)) consistently had the highest probability of selecting the GEVMAR model specification closer to the true model. Moreover, it is more probable to determine the GEVMAR specification that coincide with the true model using adjusted SNR type 2 compared to the selection of the GEVMAR specification over the true model using SNR. Finally, the most significant choice was the mixture autoregressive model using SNR type 2 and adjusted SNR, as proven by the correctness probabilities ranging from 96% to 100%.

**Table 3:** Probability of GEVMAR model (with $\text{GE}V_{I}$) based on SNR and adjusted SNR.

Model	Sample size ($\underset{,}{N}$)							
	30		50		100		500	
	P(Δ)	$P(\Delta_{2}^{*})$	P(Δ)	$P(\Delta_{2}^{*})$	P(Δ)	$P(\Delta_{2}^{*})$	P(Δ)	$P(\Delta_{2}^{*})$
GEVMAR(2;1,1)	**0.985**	**0.986**	**0.997**	**0.997**	**1**	**1**	**0.968**	**0.969**
GEVMAR(2;2,2)	0.002	0.001	0.000	0.000	0.000	0.000	0.001	0.001
GEVMAR(2;3,3)	0.000	0.000	0.000	0.000	0.000	0.000	0.000	0.000
GEVMAR(2;4,4)	0.001	0.001	0.000	0.000	0.000	0.000	0.000	0.000
GEVMAR(2;1,2)	0.008	0.008	0.003	0.003	0.000	0.000	0.015	0.015
GEVMAR(2;1,3)	0.002	0.002	0.000	0.000	0.000	0.000	0.007	0.006
GEVMAR(2;1,4)	0.002	0.002	0.000	0.000	0.000	0.000	0.009	0.009

Total	1	1	1	1	1	1	1	1

P(Δ)states probability of GEVMAR based on SNR, $P(\Delta_{2}^{*})$states probability of GEVMAR based on adjusted SNR type 2.

### Application study

#### Claim reserves data

**Fig. 1:**
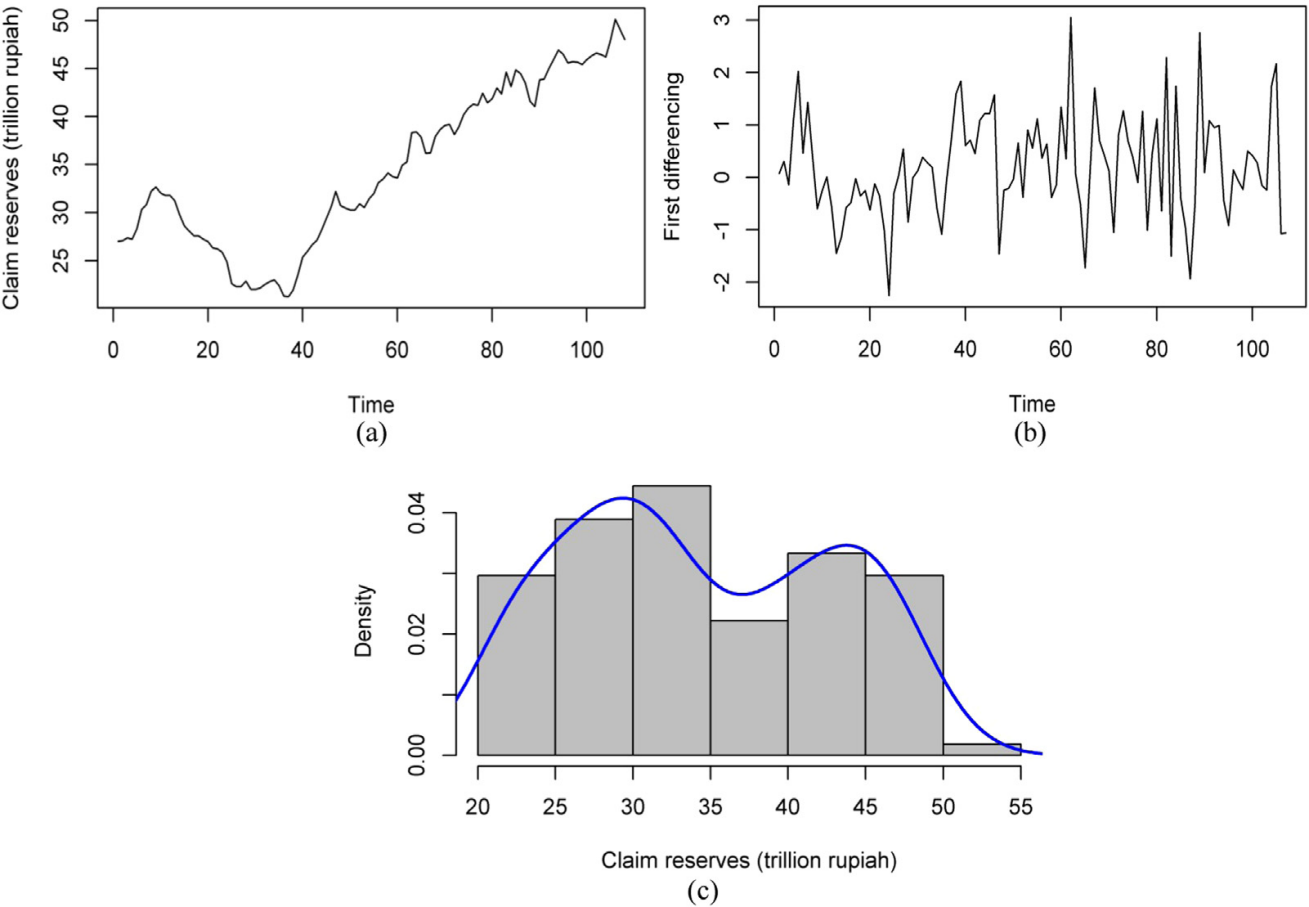
**(a)** Plot claim reserves (January 2015 to December 2023); **(b)** First differencing of claim reserves; **(c)** Marginal density of claim reserves.

This study uses data on claim reserves of general insurance in Indonesia for the period January 2015 to December 2023 sourced from Otoritas Jasa Keuangan (OJK), the Republic of Indonesia. The Bayesian GEVMAR model (with $\text{GE}V_{I}$) was used to analyze the claim reserves data of the general insurance enterprises in Indonesia, as shown by the a graphical representation of each dataset. The time series presented actuarial data, particularly claim reserves, as shown in Fig. 1 (a), while (c) is a histogram with two different modes. This requirement comprising two distinct modes at 29.453 and 42.904 was further supported by Hartigan test [37]. Fig. 1 (b) shows the visualization of the first differencing of the claim reserves. The data depicts a unique distribution pattern in the magnitude of the claim reserves value. The fit test (Table 4), particularly the Kolmogorov-Smirnov analysis, suggested that the data showed an unusual distribution pattern similar to a generalized extreme value distribution with a lower KS statistic and a higher p-value. Furthermore, the claim reserves experienced a significant decrease, from IDR 31.252 trillion in to IDR 21.231 trillion from January 2016 to 2018. This outcome was caused by the reduction in premium income and the rise in claim payments, including outstanding debts. The current uncertain economic situation tend to have an impact on the general insurance product market, potentially leading to a decline in premium income [38].

**Table 4:** Goodness of fit test for the first differencing claim reserves data.

Distribution	KS statistic	p-value
Normal (Gaussian)	0.08843	0.11734
GEV	**0.05121**	**0.61214**

**Fig. 2:**
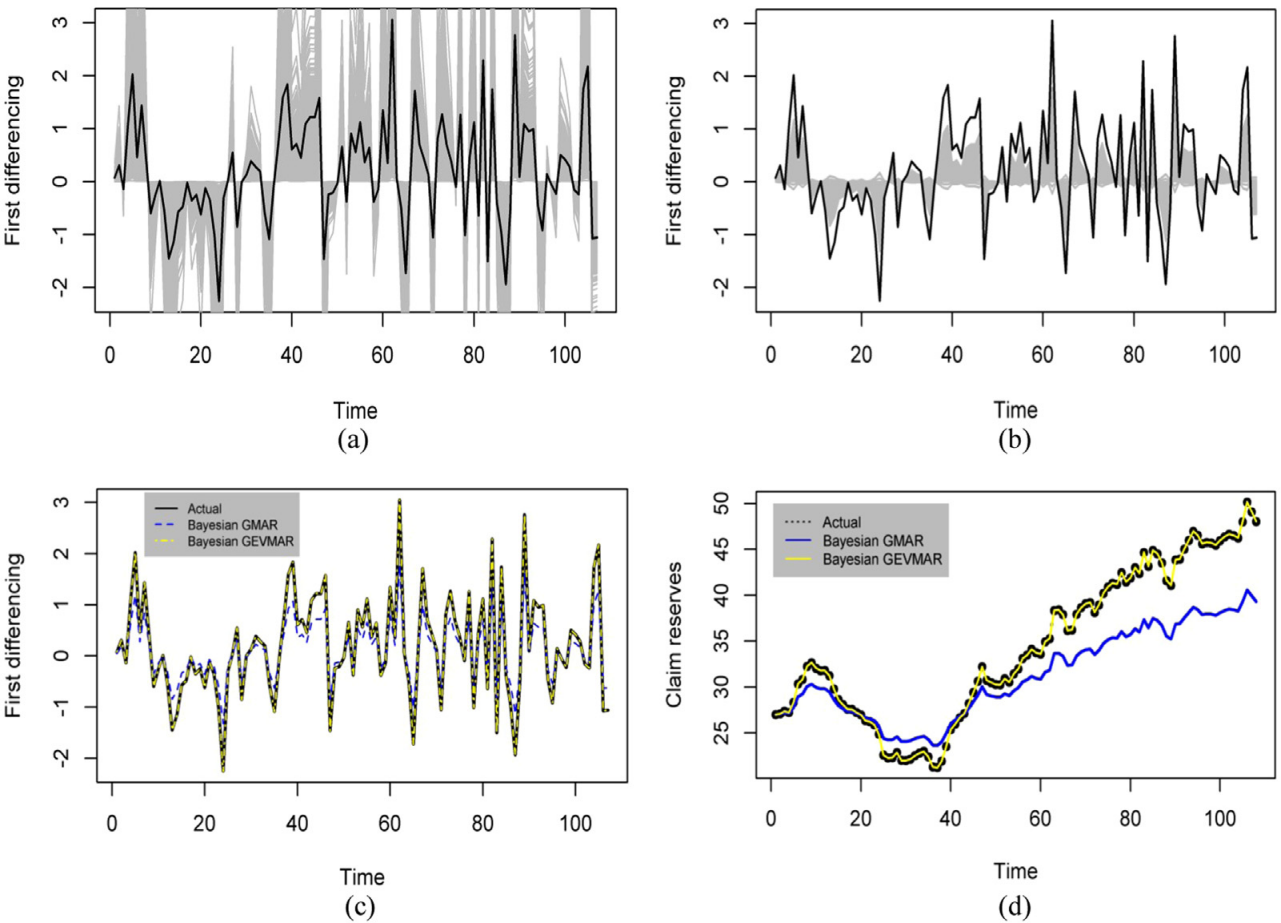
**(a)** Plot estimation and prediction of Bayesian GEVMAR(2;1,1) model; **(b)** Plot estimation and prediction of Bayesian GMAR(2;1,1) model; **(c)** Plot Prediction of the first differencing; **(d)** Plot prediction of claim reserves data with Bayesian GEVMAR(2;1,1) model and Bayesian GMAR(2;1,1) model comparison.

The estimation and prediction of the first differencing claim reserves data was conducted using the Bayesian method. Additionally, the estimated and predicted results of the GEVMAR(2;1,1), was compared to the GMAR(2;1,1) model. Fig. 2 (a) and (b) present estimation and prediction results obtained from the Bayesian GEVMAR(2;1,1) model and Bayesian GMAR(2;1,1), respectively. This points out that the Bayesian GEVMAR(2;1,1) performs better in capturing high volatility behavior than the Bayesian GMAR(2;1,1) model. This claim is further supported by how the sampling method used to estimate the posterior distribution parameters and generate predictive values results in the shaded region tracking more closely with the volatility level in the first differencing data (solid black) compared to the GMAR(2;1,1) model. This assertion is further substantiated by a supplementary piece of evidence derived from the analysis of claim reserves data, as illustrated in sections (c) and (d) of Fig. 2. The Bayesian GEVMAR(2;1,1) model (represented by the yellow and solid yellow dashed lines) demonstrates superior performance compared to the Bayesian GMAR(2;1,1) model (indicated by the blue and solid blue dashed lines), as it exhibits a closer alignment with the claim reserves data that has been empirically observed in the investigation.

The current research applied the MAPE, root mean square error (RMSE), SNR, and adjusted SNR type 2, a metric used to assess the accuracy of the model in predicting results. The subsequent section focused on the performance outcomes of the GEVMAR and GMAR models using the Bayesian method.

Based on Table 5, the GEVMAR model outperformed the GMAR. In particular, the GEVMAR(2;1,1) had superior performance compared to the other models. This was supported by the fact that the MAPE of GEVMAR(2;1,1) was 0.0189, less than the value of other models. Even when compared to GMAR(2;1,1), the difference in performance was highly substantial, with a MAPE of 7.5812. Considering this value, adjusted SNR in the GEVMAR model showed superior performance compared to the GMAR. This was supported by the adjusted SNR of GEVMAR(2;1,1), equivalent to 1.3894 × 10^6^, a much higher value compared to the adjusted SNR of other models. In addition, when comparing the adjusted SNR values of GEVMAR(2;1,1) and GMAR(2;1,1) of -1.1285, a substantial disparity was observed in the performance between the two models. Ultimately, it is evident that the GEVMAR model exhibits superior capabilities to the GMAR model in handling the volatility and multimodal characteristics of the claim reserves data. In other words, the implementation of the GEVMAR model successfully accommodates data conditions that are generally recognized to be related to long-tailed businesses [[15], [16]], such as claim reserves, which include a variety of information and significantly contribute to nonlinearity, even when presented in aggregate form [17].

**Table 5:** Performance of the GEVMAR ($\text{GE}V_{I}$) and GMAR models.

Model	RMSE	MAPE (%)	SNR	Adjusted SNR type 2
GEVMAR(2;1,1)	0.0092	**0.0189**	1.4575 × 10^6^	**1.3894 × 10^6^**
GEVMAR(2;1,2)	0.2363	0.5263	1691.7200	1607.667
GEVMAR(2;2,1)	3.8116	7.8585	3.8452	-1.3345
GEVMAR(2;2,2)	1.0452	2.1536	103.7685	93.9195
GMAR(2;1,1)	3.6936	7.5812	4.0631	-1.1285
GMAR(2;1,2)	4.3229	8.9133	2.5816	-2.5390
GMAR(2;2,1)	5.1076	10.5275	1.4456	-3.6219
GMAR(2;2,2)	5.6050	11.5569	1.0106	-4.0366

Claim reserves data, even when presented as time series still exhibited abnormal characteristics, such as extreme conditions. This greatly affected the value of claim reserves, and payments, including premium values [24]. The Bayesian method used for modeling GEVMAR was founded on this principle, particularly when applied to insurance data, highly susceptible to the impact of individual claims [9], resulting in the loss of prior information effects. It is essential to recognize that the Bayesian approach utilized in this manuscript does not emphasize the integration of prior distributions alongside the determination of hyperparameters. Nevertheless, the primary aim is to concentrate on the GEVMAR model that we advocate for the purpose of accommodating the volatility and multimodality inherent in claim reserves data via the Bayesian framework, utilizing adjusted SNR as an indicator of model efficacy. Consequently, in future endeavors, this model may also be advanced with respect to the intricate nature of claim reserves data that transcends a basic univariate model by directly involving other data such as claim payments and premium values.

## Conclusions

In conclusion, the findings showed that the GEVMAR model used GEV type I distribution and the Bayesian method to consistently regulate the adjusted SNR. The GEVMAR(2;1,1) consistently exhibited higher adjusted SNR values compared to the other model scenarios in the simulations, with regard to the sample size. By adopting the Bayesian methods, the GEVMAR showed superior performance compared to the GMAR model in the interpretation of claim reserves data. The GEVMAR(2;1,1) had adjusted SNR type 2 and MAPE values of 1.3894 × 10^6^ and 0.0189, respectively. However, the GMAR(2;1,1) had adjusted SNR type 2 value of -1.1285, which is less than the GEVMAR(2;1,1) model. The MAPE value of the GMAR(2;1,1) which was equivalent to 7.5812, significantly differed from the GEVMAR(2;1,1). Therefore, the GEVMAR was a suitable option for handling multimodal data that exhibited volatility characteristics, such as claim reserves. Additional investigation was required to verify the precision of different implementations of the GEVMAR model, particularly with regards to determining the number of components, autoregressive order, and distribution type of the generalized extreme value. In addition, the future application of more sophisticated datasets related to historical information affecting claim reserves data is regarded as critical.

## Limitations

None.

## Ethics statements

The data used in this research is secondary data derived from the official website of OJK, the Republic of Indonesia (https://www.ojk.go.id/).

## CRediT authorship contribution statement

**Chrisandi R. Lande:** Conceptualization, Methodology, Software, Writing – original draft. **Nur Iriawan:** Conceptualization, Methodology, Writing – review & editing, Supervision. **Dedy Dwi Prastyo:** Methodology, Writing – review & editing, Validation, Supervision.

## Declaration of competing interest

The authors declare that they have no known competing financial interests or personal relationships that could have appeared to influence the work reported in this paper.

## Data Availability

Data will be made available on request.
